# Évolution des Ruptures Utérines à la Maternité de L'hôpital National Ignace Deen (Chu de Conakry)

**DOI:** 10.48327/ZY14-QG95

**Published:** 2021-01-29

**Authors:** I.S. Baldé, I. Sylla, M.H. Diallo, I.T. Diallo, F.B. Diallo, A. II Sow, T. Sy, N. Keita

**Affiliations:** 1 Service de gynécologie-obstétrique de l'Hôpital national Ignace Deen, CHU de Conakry, Guinée; 2 Service de gynécologie-obstétrique de l'Hôpital national Donka, CHU de Conakry, Guinée

**Keywords:** Rupture utérine, Accouchement, Morbidité iatrogène, Pronostic maternofœtal, Mortalité périnatale, Prévention, Soins obstétricaux et néonataux d'urgence, Hôpital, Conakry, Guinée, Afrique subsaharienne, Uterine rupture, Childbirth, Iatrogenic morbidity, Maternal-fetal prognosis, Perinatal mortality, Prevention, Emergency obstetric and neonatal care, Hospital, Conakry, Guinea, Sub-Saharan Africa

## Abstract

La rupture utérine est un drame obstétrical courant de nos salles d'accouchement, devenu exceptionnel dans les pays développés. Dans les pays en développement y compris la Guinée, ce drame constitue une des préoccupations majeures de l'obstétricien. Les objectifs de ce travail étaient: d'évaluer la fréquence de la rupture utérine dans le service; de décrire les caractéristiques sociodémographiques et cliniques des patientes; d'identifier les facteurs favorisants la survenue de la rupture utérine; d'évaluer le pronostic materno-fœtal et de proposer une stratégie de prévention en vue d'une réduction de la morbidité et de la mortalité maternelle et fœtale par rupture utérine. Il s'agissait d'une étude descriptive avec recueil des données en deux phases, dont une rétrospective, d'une durée de 18 mois allant du 1er juillet 2017 au 31 décembre 2018 et l'autre prospective d'une durée de 18 mois aussi allant du 1^er^janvier 2019 au 30 juin 2020, réalisée à la maternité de l'Hôpital national Ignace Deen. Nous avons colligé 84 cas de rupture utérine sur 18790 accouchements, soit une fréquence de 0,44%. Dans le même temps 10067 césariennes ont été effectuées soit une laparotomie pour rupture utérine pour 120 césariennes. L'âge moyen des patientes était de 28,14 ans avec un écart type de 2 ans et le profil moyen était celui d'une femme au foyer (51,8%), multipare (44,6%), évacuée d'une maternité périphérique (85,5%) et ayant un nombre insuffisant de consultations prénatales (82,6%). Dans 93,14% des cas, la rupture utérine était survenue dans les maisons d'accouchements, les maternités périphériques et durant le trajet; les ruptures utérines étaient en majorité spontanées (65,1%) et survenues sur un utérus sain (59,0%). La rupture utérine était plus fréquemment complète (83,3%). Le traitement chirurgical était plus fréquemment conservateur par une hystérorraphie (88,1%). Nous avons enregistré 12 décès maternels, soit une létalité de 14,6%. Dans la quasi-totalité des cas les femmes étaient admises sans signe de vie du foetus. Pour diminuer la fréquence des ruptures utérines, il faut favoriser une meilleure organisation des soins obstétricaux et néonataux d'urgence (SONU) et un meilleur dépistage des facteurs de risque de dystocie au cours des consultations prénatales.

## Abstract (en)

Uterine rupture is a common obstetrical drama in our delivery rooms that has become exceptional in developed countries. In developing countries including Guinea, this tragedy is one of the major concerns of the obstetrician. The objectives of this work were: to evaluate the frequency of uterine rupture in the department, to describe the socio-demographic and clinical characteristics of the patients, to identify the factors favoring the occurrence of uterine rupture, to evaluate the maternal-fetal prognosis and propose a prevention strategy to reduce maternal and fetal morbidity and mortality by uterine rupture. This was a descriptive study with data collection in two phases, one retrospective lasting 18 months from July 1, 2017 to December 31, 2018 and the other prospective, lasting 18 months also from January 1, 2019 to June 30, 2020 both carried out at the maternity ward of the Ignace Deen National Hospital. We collected 84 cases of uterine rupture out of 18,790 deliveries, i.e. a frequency of 0.44%. During the same time 10,067 cesarean sections were realized, i.e. one laparotomy for uterine rupture for 120 cesarean sections. The average age of the patients was 28.14 years with a standard deviation of 2 years and the average profile is that of a housewife (51.8%), multiparous (44.6%), evacuated from peripheral maternity (85.5%) and having an insufficient number of antenatal consultations (82.6%). In 93.1% of cases, the uterine rupture had occurred in delivery centers, peripheral maternity hospitals and on the way, the uterine ruptures were mostly spontaneous (65.1%), and occurred in a healthy uterus (59.0%). Uterine rupture was more frequently complete (83.33%). Surgical treatment was more frequently conservative with hysterorrhaphy (88.1%). We recorded 12 maternal deaths, i.e. a case fatality rate of 14.6%. On admission, almost all of the women showed no signs of fetal life. To reduce the frequency of uterine ruptures, better organization of emergency obstetric and neonatal care and better screening for risk factors for obstructed labor during prenatal consultations should be encouraged.

## Introduction

La santé maternelle est une préoccupation majeure à cause du ratio de la mortalité maternelle qui reste toujours élevé en Guinée (550 décès pour 100000 naissances vivantes) [6], très largement supérieur à ceux des pays développés où il varie de 5 à 30 décès maternels pour 100000 naissances vivantes [17] et ce malgré les progrès importants enregistrés dans le cadre du programme national de réduction de la mortalité maternelle. La femme guinéenne continue ainsi à payer un lourd tribut aux complications liées à la grossesse et à l'accouchement.

La mortalité maternelle demeure ainsi un véritable problème de santé publique dans nos pays en voie de développement alors que 80% des décès maternels seraient évitables selon l'OMS [17].

Parmi les complications obstétricales en cause dans les décès maternels, la rupture utérine est certainement l'une des plus graves, car mettant en jeu immédiatement le pronostic vital maternel et fœtal, constituant un drame obstétrical courant de nos salles d'accouchement en pays à ressources limitées, témoignant d'une mauvaise qualité des soins obstétricaux et par conséquent, constituant un besoin obstétrical non couvert.

La rupture utérine est un accident obstétrical grave caractérisé par la présence d'une solution de continuité non chirurgicale, partielle ou complète d'un utérus gravide [9].

Les facteurs favorisants de la rupture utérine sont liés à une mauvaise surveillance du travail d'accouchement, une insuffisance de personnel qualifié et des conditions socioéconomiques défavorables [15]. Elle fait partie des urgences obstétricales que nous rencontrons dans nos pratiques quotidiennes à la maternité de l'Hôpital national Ignace Deen.

Trois études antérieures, réalisées en Guinée en 1991 et 1998 à la maternité de l'Hôpital Ignace Deen et en 2014 à la maternité Donka avaient rapporté des fréquences respectives de 0,41% des accouchements, 14,28% des causes d'hémorragies obstétricales qui sont les premières causes obstétricales directes de décès maternels et 0,36% des accouchements [2, 4, 7].

La fréquence élevée de cette pathologie et de la mortalité maternelle et fœtale qu'elle entraîne, les difficultés liées à sa prise en charge et l'intérêt de l'amélioration de la qualité des soins obstétricaux et néonataux d'urgence (SONU) ont motivé plus de 20 ans après, la réalisation de la présente étude afin d'évaluer l'évolution de ce problème. Le but de la présente étude était de contribuer à l'étude de la rupture utérine. Il s'agissait plus spécifiquement:

- d'évaluer la fréquence de la rupture utérine dans le service et la comparer aux fréquences antérieures;
- de décrire les caractéristiques sociodémographiques et cliniques des patientes (Tableaux I et II);
- d'identifier les facteurs favorisants la survenue de la rupture utérine;
- d'évaluer le pronostic materno-fœtal;
- et de proposer une stratégie de prévention en vue d'une réduction de la morbidité et de la mortalité maternelle et fœtale par rupture utérine.

**Tableau I:** Répartition des patientes selon leurs caractéristiques sociodémographiques Distribution of patients according to their socio-demographic characteristics

Caractéristiques sociodémographiques	Nombre	%
**Tranche d'âge**		
16-21	11	13,1
22-27	17	20,2
28-33	30	35,7
34-39	17	20,2
40 et plus	9	10,8
minimum = 17	moyenne = 28,14	maximum = 47
**Profession**		
élève/étudiante	5	6,0
femme au foyer	43	51,2
libérale	30	35,7
salariée	6	7,1
**Provenance**		
Kaloum	4	4,8
Dixinn	5	6,0
Matam	10	11,9
Ratoma	29	34,5
Matoto	29	34,5
hors Conakry	7	8,3
**Niveau d'instruction**		
non scolarisée	52	61,9
primaire	8	9,5
secondaire	16	19,1
universitaire	8	9,5
**Parité**		
multipare	37	44,0
paucipare	29	34,5
primipare	18	21,5
**Mode d'admission**		
évacuée	71	84,5
venue d'elle-même	13	15,5
**Nombre de CPN**		
0	2	2,5
1-2	23	27,4
3-4	36	42,8
> 4	23	27,4

CPN (Consultation prénatale)

**Tableau II:** Répartition des patientes selon les caractéristiques cliniques Distribution of patients according to clinical characteristics

	Nb	%
**Lieu de survenue**		
domicile	9	10,7
maternité périphérique	53	63,0
service	5	6,0
trajet	17	20,3
**Moment de survenue**		
grossesse	8	9,5
travail	76	90,5
**Circonstances étiologiques**		
spontanée	54	64,2
iatrogène	30	35,8
ocytocine	12	14,3
prostaglandine	10	12,0
expression abdominale	6	7,1
traumatisme	2	2,4
**Tableau clinique**		
diagnostic rétrospectif	19	22,6
rupture franche	65	77,4

## Méthodologie

Il s'agissait d'une étude descriptive réalisée à la maternité de l'Hôpital national Ignace Deen (CHU de Conakry) avec recueil des données en deux phases, dont une rétrospective d'une durée de 18 mois allant du 1^er^ juillet 2017 au 31 décembre 2018 et l'autre prospective d'une durée de 18 mois allant du 1er janvier 2019 au 30 juin 2020.

Toutes les gestantes et parturientes qui ont présenté une rupture utérine ont été incluses dans l'étude.

N'étaient pas incluses dans l'étude les cas de déchirures du col et les perforations utérines d'origine traumatiques.

Pour chaque patiente, les paramètres suivants ont été étudiés: l'âge maternel, la profession, la zone de provenance, la parité, l'état de l'utérus (cicatriciel ou sain), le mode d'admission, le nombre de consultations prénatales (CPN), le lieu de survenue de la rupture, les circonstances de découverte, les facteurs favorisants, le type et le siège des lésions, les lésions associées, la prise en charge, le poids du nouveau-né, la durée de l'intervention chirurgicale, les complications post opératoires, la durée d'hospitalisation et la létalité maternelle et fœtale (Tableau III).

**Tableau III:** Répartition des patientes selon la prise en charge Distribution of patients according to management

Caractéristiques	Nombre	%
**Réanimation**		
oui	64	76,2
non	20	23,8
**Transfusion**		
oui	51	60,7
non	33	39,3
**Durée d'intervention (min)**		
< 30	3	3,6
30-60	47	55,9
> 60	31	36,9
minimum = 15	moyenne = 53,4	maximum = 128
**Durée d'hospitalisation (jours)**		
< 3	8	9,5
3-6	67	79,8
> 6	6	7,1
minimum = 1	moyenne = 6,1	maximum = 61
**Technique chirurgicale**		
hystérectomie	10	11,9
hystérorraphie	71	8,1

La technique de collecte était basée sur l'extraction des données à partir des dossiers pour la partie rétrospective (revue documentaire), sur l'interview au lit de la malade, l'observation des malades pour la partie prospective.

L'analyse des données a été faite par le logiciel Epi info.

## Résultats

Durant la période d'étude, nous avons enregistré 84 cas de rupture utérine pour 18790 accouchements réalisés dans le service, soit une fréquence de 0,44%; on dénombrait dans la même période 10067 césariennes, soit une fréquence de 0,83% (une rupture utérine versus 120 césariennes).

La majorité des ruptures sont survenue sur un utérus sain (non cicatriciel) soit 59% contre 41% d'utérus cicatriciel.

Le siège de la rupture était essentiellement segmento-corporéal dans 70,2% des 81 cas opérés et segmentaire dans 26,2%.

Dans 83,3% des cas, la rupture était complète et dans 16,7% des cas incomplète (sous-péritonéale).

Dans la plupart des cas (66,8%), il n'y avait pas de lésions associées; cependant, on a noté une extension de la rupture au col de l'utérus dans 17 cas (21%), à la vessie dans sept cas (8,6%), au pédicule utérin dans trois cas (3,7%), au ligament large et au colon sigmoïde dans un cas (1,2%).

Sur les 81 cas opérés, nous avons enregistré 65,4% de suites opératoires compliquées contre 34,6% de suites non compliquées. Les complications étaient réparties comme suit: 41 cas d'anémies (48,8%), 14 cas d'état de choc (21,4%) dont 12 cas de choc hémorragique (14,3%), deux cas de choc infectieux (2,4%) huit cas d'endométrite (9,5%), sept cas de suppurations pariétales (8,3%).

Nous avons enregistré 12 cas de décès maternels par rupture utérine, soit une létalité de 14,6% et un ratio de 75,41 décès maternels par rupture utérine pour 100000 naissances vivantes.

Parmi les 12 décès, 3 sont survenus en préopératoires, 6 en peropératoires et 3 en post opératoires au cours de l'hospitalisation.

Nous avons enregistré 14,2% de foetus macrosomes contre 85,8% de foetus de poids normal. Dans la quasi-totalité des cas, 84,3% les femmes étaient admises sans signes de vie du foetus contre 15,7% de nouveau-nés qui ont survécu.

## Discussion

Les ruptures utérines spontanées surviennent pendant le travail en cas de dystocie maternelle ou fœtale [8]. Dans notre étude, la majorité des ruptures utérines (90,5%) sont survenues pendant le travail, le plus souvent sur utérus sain (59,0%): constat partagé par Koné et al rapportant qu'en Afrique noire 45 à 85% des ruptures utérines ne surviennent que sur des utérus sains [11]. Par contre, dans les pays industrialisés, la principale cause des ruptures utérines est la désunion cicatricielle: Fedorkowa et al [8] au Canada dans une série de 15 ruptures utérines ont rapporté sept cas de rupture utérine par désunion de cicatrice de césarienne.

La fréquence plus élevée de ruptures utérines complètes est en rapport avec le mode d'admission de nos patientes qui pour la plupart, étaient évacuées des maternités périphériques après un séjour de plusieurs heures, voire plusieurs jours, par un personnel non qualifié ignorant les contre-indications de l'accouchement par voie basse et abusant de l'usage des ocytociques. Les victimes sont donc pour la plupart évacuées d'une maternité périphérique (84,5%), dans un rayon de plus de 50 km pour certaines patientes (villes limitrophes de Conakry), dans des conditions souvent précaires (transport non médicalisé), donnant le temps à la rupture de se produire avant l'arrivée au centre de référence.

Le pronostic maternel est marqué par une létalité maternelle élevée (14,6%), qui a certes considérablement baissé comparativement à celle rapportée par Keita et al [10] à la maternité soeur de l'Hôpital national Donka en 1989 soit 25,2%. Notre taux de décès maternel est inférieur à celui rapporté par Ngbale et al [12] en République centrafricaine (21,4%): il est par contre supérieur à celui rapporté par Zine et al [17] en Tunisie (2,8%). Cette mortalité pourrait être améliorée par la politique de décentralisation des SONU.

Le pronostic fœtal est resté également péjoratif avec un taux de mortinatalité de 84,3+%, taux superposable à ceux de Ngbale et al [12] (83%) et Sépou et al [14] (80%) en République centrafricaine, taux par contre inférieur à celui de Cissé et al [1] au Sénégal (94%).

La moyenne d'âge est comparable à celle rapportée par Diallo et al [4] (28,3 ± 2 ans) et Ngbale et al [12] (28,3 ± 2 ans); elle est par contre supérieure à celle rapportée par Diallo et al [3] au Niger (44% entre 21 et 35 ans avec une moyenne de 26 ans), et inférieure à celles rapportées par Vangreenderhysen et al [16] au Niger (29 ans) et Cisse et al [1] au Sénégal (31 ans ± 2 ans). Cela dénote un recul de l'âge de survenue de cet accident chez les parturientes qui peut s'expliquer, d'une part par la fréquence non négligeable de grossesses précoces dans nos régions, elles-mêmes corolaires des mariages précoces en Afrique et, d'autre part la fréquence non négligeable de jeunes femmes multipares.

Les femmes au foyer ont payé le plus lourd tribut avec 51,1% des patientes, suivies par les femmes ayant une profession libérale (35,7%). L'analphabétisme et le bas niveau socioéconomique sont identifiés par la plupart des auteurs comme facteurs prédisposants à la rupture utérine [5, 13].

Les multipares étaient les plus touchées avec 44,0%, la multiparité étant un principe de procréation ancré dans nos sociétés à bas niveaux d'éducation et d'information, sans compter les difficultés à accéder aux soins de planification familiale. Cette fréquence élevée de paucipares et de multipares peut s'expliquer par la précocité du mariage en Afrique et la grande multiparité des femmes souvent jeunes [3].

La fréquence de la rupture utérine n'a pas considérablement changé au cours de ces trois dernières décennies, comparativement à celle rapportée par Diallo et al [7] dans le même service en 1991 soit 0,41% et celle rapportée par Diallo et al [4] dans la maternité soeur de l'Hôpital national de Donka en 2014 soit 0,36%. La fréquence a par contre considérablement baissé par rapport à celle rapportée par Keita et al [10] en 1989 dans la maternité soeur de l'Hôpital national Donka soit 0,74% de l'ensemble des accouchements et 12,76% des accouchements par césarienne (une rupture utérine pour 20 césariennes).

La fréquence toujours élevée de 0,44% de ruptures utérines par rapport au nombre d'accouchements durant la même période dans notre série est voisine de celles rapportées par divers auteurs africains, notamment, Ngbale et al [12] en République centrafricaine, (0,3%) et Kone et al [11] en 1995 (0,13 à 3,3% en Afrique noire). Les pays africains partagent en général plusieurs caractéristiques (sous équipements des services, bas niveaux socio-économiques des populations, insuffisance de personnel de santé qualifié…).

Cette fréquence élevée pourrait aussi s'expliquer par la non-fonctionnalité de la maternité soeur de l'hôpital national Donka en raison de travaux de rénovations depuis juillet 2015, surchargeant depuis la maternité de l'Hôpital national d'Ignace Deen devenu le seul service de référence des maternités de la ville de Conakry prenant en charge toutes les parturientes présentant un travail dystocique provenant du domicile ou des maternités périphériques. Cette fréquence pourrait être réduite si les accouchements compliqués étaient distribués entre les deux services grâce à l'augmentation du nombre de personnel qualifié au sein du service (qui est l'une des deux maternités de référence du pays) et des centres de santé (maternités périphériques) et à l'amélioration de l'aptitude du personnel des maternités périphériques à diagnostiquer et à prendre en charge ou évacuer en temps opportun les urgences obstétricales vers les services de référence grâce aux divers ateliers de formation en SONU organisés sur l'ensemble du territoire.

Les communes de Ratoma et de Matoto, 34,5% chacune, étaient les plus grandes pourvoyeuses de rupture utérine. Ces 2 communes bien qu'étant les plus peuplées de Conakry ne disposent que d'un seul service de SONU complets avec une capacité très limitée. Les maternités communales de la ville de Conakry disposent d'un bloc opératoire souvent non fonctionnel la nuit, ce qui contraint les praticiens hospitaliers de ces communes à évacuer vers le service de référence la majeure partie des patientes chez qui une césarienne est indiquée. On note également une prolifération anarchique des maisons d'accouchement tenues par des personnes non qualifiées (agent de santé à la retraite, garçons de salles, matrones) où les patientes séjournent longtemps, recevant toutes sortes de traitement et des manoeuvres inadaptées avant d'être évacuées.

Dans notre série, les causes iatrogènes non négligeables de cet accident sont la conséquence du non-respect des principes et techniques de l'obstétrique moderne, notamment, l'usage inconsidéré des ocytociques et prostaglandines par le personnel pour accélérer le travail (perfusion abusive et injection intra-murale d'ocytocine, non-respect des modalités d'administration, indications et contre-indications du misoprostol), pressions abdominales à dilation complète en cas d'efforts expulsifs insuffisants et traumatismes consécutifs à certaines manoeuvres obstétricales (version par manoeuvre interne).

Ces ruptures pourraient être évitées par un meilleur suivi du partogramme, le respect des principes et technique de l'obstétrique moderne dans la gestion du travail d'accouchement et surtout l'impératif d'évacuation sur les maternités des hôpitaux de référence lors de la dernière consultation prénatale (CPN) en cas de facteur de risque identifié par rapport à l'évacuation obstétricale d'urgence. Notre constat est identique à celui de Rabarikoto et al [13] à Madagascar, rapportant que la rupture utérine survenait majoritairement chez des patientes n'ayant pas effectué de CPN (72,2%). Il se pose le problème de la décentralisation de la prise en charge des dystocies.

Malgré la gratuité des SONU instaurée dans la majeure partie des pays d'Afrique subsaharienne, les femmes préfèrent consulter dans les maisons d'accouchement probablement du fait de la proximité géographique, de l'aspect social et culturel ainsi que de la qualité de l'accueil et de l'hospitalité, bien qu'il s'agisse de structures de santé illégales. Ainsi, 63,0% ont rompu leurs utérus dans les maisons d'accouchements et maternités périphériques, 10,7% à domicile et seulement cinq cas sont survenues dans le service. C'est après une longue tentative infructueuse d'accouchement par voie basse que les patientes sont adressées dans notre service pour une prise en charge. Elles arrivent dans le service dans un tableau de rupture utérine constitué. En outre, elles ne bénéficient d'aucune assistance médicale correcte avant et durant leur transfert.

L'urbanisation illégale et l'extension anarchique de la ville de Conakry, à l'instar des autres capitales africaines, aggravent la mauvaise distribution de la carte sanitaire, rendant ainsi difficile l'accès des patientes aux structures fournissant des services de qualité.

Nous avons fait appel à la réanimation rapide et intense, concomitante de la chirurgie dans 76,2% des cas: cette chirurgie a été le plus souvent conservatrice par hystérorraphie (88,1%). Cette attitude conservatrice se retrouve chez plusieurs auteurs de pays africains [7, 17]. La chirurgie conservatrice est l'une de nos préoccupations en cas de rupture utérine. Chez les patientes de moins de 40 ans présentant des lésions utérines peu étendues et non septiques, multipares ne consentant pas à l'hystérectomie, l'hystérorraphie associée à la ligature des trompes a été pratiquée. Par rapport à notre taux d'hystérectomie (11,9%), nous avons tenu compte de certains paramètres à savoir: l'âge, la parité, l'étendue des lésions, l'atteinte des pédicules vasculaires, l'état du muscle utérin et la présence ou non d'infection.

## Conclusion

La fréquence des ruptures utérines reste toujours non négligeable dans le service (0,44%) avec une mortalité maternelle et fœtale élevées: c'est ainsi un drame obstétrical courant de nos salles d'accouchements, témoins d'une mauvaise qualité des soins obstétricaux et par conséquent d'un besoin obstétrical non couvert.

Ces ruptures utérines pourraient être évitées dans leur quasi-totalité par: une sensibilisation des femmes enceintes, une amélioration de la gestion de l'urgence obstétricale, et en rendant les consultations prénatales plus opérationnelles en vue d'un meilleur dépistage des facteurs de risques obstétricaux. C'est pour cette raison qu'il faut mettre l'accent sur la prévention.

La décentralisation et la prise en charge appropriée de l'urgence obstétricale est à encourager. Il faut insister sur la nécessité de faire un dépistage sérieux des dystocies et leur prise en charge appropriée par l'amélioration de l'accessibilité à la césarienne, le respect des indications obstétricales de césarienne et la prise en charge des patientes à haut risque de rupture utérine.

## Conflit D'intérêt

Les auteurs ne déclarent aucun conflit d'intérêt.
